# Mouse models of sporadic thyroid cancer derived from BRAF^V600E^ alone or in combination with PTEN haploinsufficiency under physiologic TSH levels

**DOI:** 10.1371/journal.pone.0201365

**Published:** 2018-08-07

**Authors:** Mika Shimamura, Nobuyuki Shibusawa, Tomomi Kurashige, Zhanna Mussazhanova, Hiroki Matsuzaki, Masahiro Nakashima, Masanobu Yamada, Yuji Nagayama

**Affiliations:** 1 Department of Molecular Medicine, Atomic Bomb Disease Institute, Nagasaki University, Nagasaki, Japan; 2 Department of Medicine and Molecular Science, Graduate School of Medicine, Gunma University, Maebashi, Japan; 3 Department of Tumor and Diagnostic Pathology, Atomic Bomb Disease Institute, Nagasaki University, Nagasaki, Japan; Universidade do Porto Faculdade de Medicina, PORTUGAL

## Abstract

The BRAF^V600E^ mutation is the most prevalent driver mutation of sporadic papillary thyroid cancers (PTC). It was previously shown that prenatal or postnatal expression of BRAF^V600E^ under elevated TSH levels induced thyroid cancers in several genetically engineered mouse models. In contrast, we found that postnatal expression of BRAF^V600E^ under physiologic TSH levels failed to develop thyroid cancers in conditional transgenic *Tg(LNL-Braf*^*V600E*^*)* mice injected in the thyroid with adenovirus expressing Cre under control of the thyroglobulin promoter (Ad-TgP-Cre). In this study, we first demonstrated that *Braf*^*CA/+*^ mice carrying a Cre-activated allele of *Braf*^*V600E*^ exhibited higher transformation efficiency than *Tg(LNL-Braf*^*V600E*^*)* mice when crossed with *TPO-Cre* mice. As a result, most *Braf*^*CA/+*^ mice injected with Ad-TgP-Cre developed thyroid cancers in 1 year. Histologic examination showed follicular or cribriform-like structures with positive TG and PAX staining and no colloid formation. Some tumors also had papillary structure component with lower TG expression. Concomitant PTEN haploinsufficiency in injected *Braf*^*CA/+*^*;Pten*^*f/+*^ mice induced tumors predominantly exhibiting papillary structures and occasionally undifferentiated solid patterns with normal to low PAX expression and low to absent TG expression. Typical nuclear features of human PTC and extrathyroidal invasion were observed primarily in the latter mice. The percentages of pERK-, Ki67- and TUNEL-positive cells were all higher in the latter. In conclusion, we established novel thyroid cancer mouse models in which postnatal expression of BRAF^V600E^ alone under physiologic TSH levels induces PTC. Simultaneous PTEN haploinsufficiency tends to promote tumor growth and de-differentiation.

## Introduction

Sporadic thyroid cancers usually develop via abnormal activation of the RAS-RAF-MEK-ERK signaling pathway (MAPK; which relays signals from cell membrane to nucleus), primarily as a result of point mutations in the *RAS/BRAF* genes or chromosomal rearrangements such as *RET/PTC* translocations [1]. In the *BRAF* gene, the T1799A transverse point mutation results in a mutant BRAF, BRAF^V600E^, which exhibits constitutive serine/threonine kinase activity.

The carcinogenicity of BRAF^V600E^ in the thyroid glands was first demonstrated *in vivo* in *Tg-Braf*^*V600E*^ transgenic mice expressing BRAF^V600E^ under control of thyroid-specific thyroglobulin (*Tg*) promoter; these mice developed thyroid cancers very early in life [2]. However, this model had various limitations, including (i) BRAF^V600E^ was expressed in all thyroid cells from the fetal period, suggesting that this is a model of hereditary rather than sporadic thyroid cancers; (ii) serum TSH levels were elevated by BRAF^V600E^-mediated suppression of thyroid function, which by itself can induce thyroid goiters and sometimes tumors; and (iii) BRAF^V600E^ expression was controlled by the *Tg* promotor rather than the original *Braf* promoter [3]. These limitations remained unsolved in subsequent mouse models of thyroid cancer. *LSL-Braf*^*V600E*^*;TPO-Cre* mice expressed BRAF^V600E^ in all the thyroid cells from the fetal period, with ~8- to 80-fold increases in TSH, although TSH was expressed at physiologic levels under the control of the chromosomal promoter [4]. *Braf*^*CA*^*;Thyro*::*CreER* mice were generated to control expression of BRAF^V600E^ by tamoxifen in the postnatal period, but untreated mice displayed increased thyroid volumes 1 month after birth, presumably due to aberrant nuclear localization of CreER^T2^ in the absence of tamoxifen [5]. In that model, *Braf*^*CA*^ mice carried a Cre-activated allele of *Braf*^*V600E*^ [6], similar to *LSL-Braf*^*V600E*^ mice mentioned above [7]. Leakiness of CreER in the absence of tamoxifen has also been reported [8]. *Tg-rtTA/tetO-Braf*^*V600E*^ mice expressed BRAF^V600E^ in all the thyroid cells, with >100-fold increases in TSH, although expression began after birth (after administration of doxycycline) [9]. Finally, *Braf*^*CA*^*;TPOCreER* mice were reported to develop thyroid cancers after birth (after administration of tamoxifen), although TSH increased slightly (<10-fold) [10].

To establish an ideal mouse model of sporadic thyroid cancer, we previously generated *Tg(LNL-Braf*^*V600E*^*)* mice. Upon injection of adenovirus expressing Cre under control of the *Tg* promoter (Ad-TgP-Cre) into their left thyroid lobes at age of ~4 weeks, these mice expressed BRAF^V600E^ in a fraction of the thyroid cells. As such, serum TSH remained within physiologic range, and mice did not develop thyroid cancer [3]. From these data, we concluded that postnatal expression of BRAF^V600E^ alone in a small number of thyroid cells under normal TSH levels is insufficient for thyroid cancer development. However, this model also had a drawback; a comparison of data from the previous reports [3, 4] suggested that Cre-mediated DNA recombination was less efficient in *Tg(LNL-Braf*^*V600E*^*);TPO-Cre* mice than *LSL-Braf*^*V600E*^*;TPO-Cre* mice, as serum TSH levels increased in the latter not the former.

In the present study, therefore, we first confirmed the higher transformation efficiency of Cre-mediated DNA recombination in *Braf*^*CA*^*;TPO-Cre* mice compared with *Tg(LNL-Braf*^*V600E*^*);TPO-Cre* mice in our laboratory and then used *Braf*^*CA*^ mice rather than *Tg(LNL-Braf*^*V600E*^*)* mice to re-evaluate the carcinogenesis of BRAF^V600E^ in the context of our experimental setting with Ad-TgP-Cre. Here, we show that postnatal BRAF^V600E^ expression alone under physiologic TSH levels is sufficient for thyroid cancer development. In addition, we also studied the effect of concomitant PTEN haploinsufficiency on BRAF^V600E^-induced thyroid cancers and show that the simultaneous reduction of PTEN expression tends to promote tumor growth and de-differentiation. Our results also demonstrate development of thyroid hyperplasia/adenoma in *Pten*^*Δ/+*^ mice (but not *Pten*^*f/+*^ mice) injected with Ad-TgP-Cre, suggesting that the timing of PTEN reduction (*i*.*e*., prenatal *vs*. postnatal) is critical for tumorigenicity of PTEN in the thyroid.

## Materials and methods

### Mice used

Conditional transgenic *Braf*^*V600E*^ mice (*Tg(LNL-Braf*^*V600E*^*)#213MM*) and *TPO-Cre* mice were previously described [3, 11]. *Braf*^*CA*^ (B6.129P2(Cg)-*Braf*^*tm1Mmcm*^/J, stock# 017837) mice [6] were obtained from Jackson Laboratory. *Pten*^**Δ/+**^ mice were obtained from National Cancer Institute at Frederick, MD, USA) [12, 13]. All mice were of a B6 genetic background, except *TPO-Cre*, which were FVB/NCr.

All mice were kept in a specific pathogen-free facility. Animal care and all experimental procedures were performed in accordance with the Guideline for Animal Experimentation of Nagasaki University with approval of the Institutional Animal Care and Use Committee (permission number: 1309021089). All surgeries were performed under isoflurane anesthesia, and every effort was made to minimize suffering.

### Adenovirus used

Ad-TgP-Cre was used in this study, as described previously [3].

### Experimental designs

Surgery and injection of adenovirus into the left lobe of the thyroid of ~4-week-old mice were performed as described previously [3]. A total of 3~4 x 10^9^ adenovirus particles/mouse were injected. The number of mice in each group was shown in Table 1 (n = 5~13). The male to female ratio was approximately 1:1 in all the experimental groups. No mice died during the experimental period. After 6 months and 1 year, mice were anesthetized with isoflurane, blood was collected via cardiac tap for serum preparation, and the animals were euthanized by cervical dislocation. For histological examinations, thyroid was removed from all the mice, and lungs were removed when macroscopically visible nodules were observed (2 *Braf*^*thyr-V600E*^ and 6 *Braf*^*thyr-V600E*^*;Pten*^*thyr-Δ/+*^ mice).

**Table 1:** Summary of the results.

Mice	Adenovirus	Observation periods (weeks)	Thyroid pathology		
			Normal	Hyperplasia /adenoma	Cancer
*Braf*^*CA/+*^	-	52	5/5	0	0
*Braf*^*CA/+*^	Ad-TgP-Cre	26	9/9	0	0
*Braf*^*CA/+*^	Ad-TgP-Cre	52	1/9	0	8/9
*Braf*^*CA/+*^*;Pten*^*f/+*^	Ad-TgP-Cre	52	0	0	9/9
*Pten*^*f/+*^	Ad-TgP-Cre	52	7/7	0	0
*Pten*^*Δ/+*^	-	26~33*	2/13	11/13	0

*, *Pten*^*Δ/+*^ mice were sacrificed at 6–8 months old because of tumor development in other organs.

### H & E staining and immunohistochemistry

Tissues were fixed in 10% neutral-buffered formalin and then embedded in paraffin. Sections (4-μm-thick) were prepared and stained with hematoxylin eosin (H & E) or immunostained with primary antibody: rabbit polyclonal anti-surfactant protein A (ab115791, Abcam, Cambridge, UK; dilution of 1:500), rabbit monoclonal anti-PTEN (D4.3, Cell Signaling, Danvers, MA; dilution of 1:25), rabbit polyclonal anti-PAX8 (Pan-PAX, 21383-1-AP, Proteintech, Japan, Tokyo; dilution of 1:1,500), mouse monoclonal anti-BRAF^V600E^ (VE1, Spring Bioscience, Pleasanton, CA; dilution of 1:100), rabbit monoclonal anti-Ki-67 (ab66155, Abcam; dilution of 1:100), rabbit monoclonal anti-thyroglobulin (ab156008, Abcam; dilution of 1:250) or rabbit monoclonal anti-phospho-p44/42 MAPK (ERK1/2) (#4370S, Cell Signaling; dilution of 1:200). It should be noted here that the protein recognized by anti-PAX8 mentioned above is called "PAX" throughout the paper, because, although the immunogen for this antibody was a part of human PAX8 (212 amino acids), its specificity to PAX8 has not been confirmed. The primary antibody was followed by incubation with secondary antibody: swine anti-rabbit IgG/HRP (P0399, DAKO, Glostrup, Denmark; dilution of 1:50) or rabbit anti-mouse IgG/HGRP (PO260, DAKO; dilution of 1:100). Color was developed with 3, 3’-diaminobenzidine substrate. Slides were analyzed using an All-in-One BZ-9000 Fluorescence Microscope (Keyence, Osaka, Japan). A total of 1,500 cells were evaluated to determine the percentage of Ki67-positive cells.

### Evaluation of apoptosis

Terminal deoxynucleotidyl transferase (TdT)-mediated dUTP nick end-labeling (TUNEL) was performed with the Apop-tag™ Fluorescein Direct in situ apoptosis detection kit (Merck Millipore, Darmstadt, Germany). Slides were embedded with VECTASHIELD mounting medium containing DAPI (Vector Laboratories, Burlingame, CA) and analyzed using an All-in-One BZ-9000 Fluorescence Microscope (Keyence). A total of 1,500 cells were evaluated in each sample to determine the percentage of TUNEL-positive cells.

### Serum TSH measurements

Serum TSH was measured using a specific mouse TSH RIA with mouse TSH/LH reference (AFP9090D), mouse TSH antiserum (AFP98991) and rat TSH antigen (NIDDK-rTSH-I-9) as described previously [3, 14]. The normal range was defined as the mean ± 3 S.D. of control untreated mice.

### Statistical analyses

All data were analyzed for significant differences using the Student’s *t*-test. A p-value of less than 0.05 was considered statistically significant.

## Results

**Fig 1:**
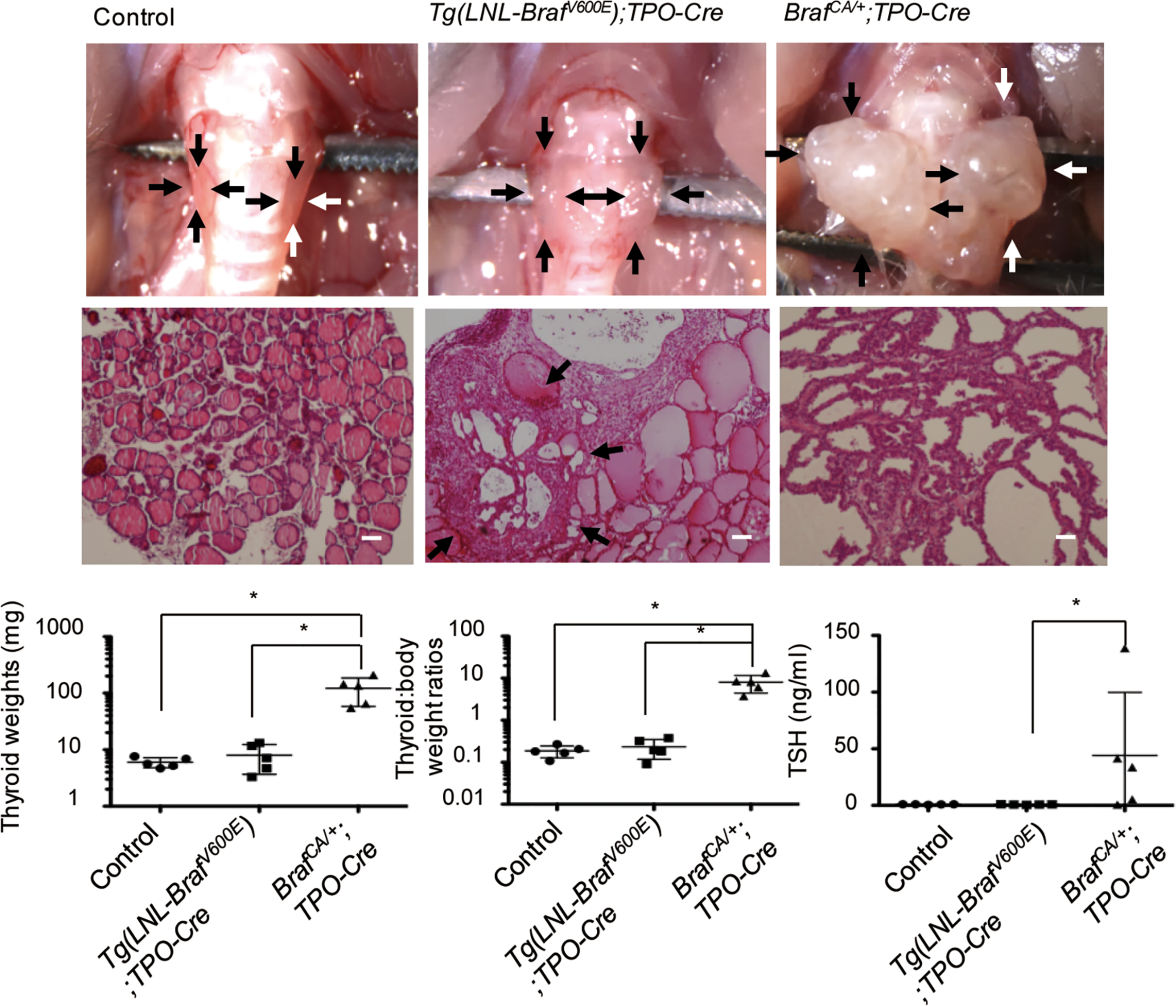
Gross appearance, histology, thyroid weight, thyroid weight to body weight ratio, and serum TSH concentration in control, *Tg(LNL-Braf*^*V600E*^*)#213MM;TPO-Cre* mice and *Braf*^*CA/+*^*;TPO-Cre* mice. Mice were sacrificed at 12 weeks of age. The thyroid gland was removed, serum was collected, and thyroid weight and TSH concentration were determined as described in the Materials and Methods. The thyroid gland and a focus of cell proliferation are indicated by arrows. *, p < 0.01. Scale bars, 50 μm.

In previous research reported by us [3] and others [4], *Tg(LNL-Braf*^*V600E*^*)****#****213MM* (a high expressor)*;TPO-Cre* mice exhibited a slightly (but not significantly) enlarged thyroid with focal neoplastic lesions and normal TSH levels, whereas *Braf*^*CA/+*^*;TPO-Cre* mice exhibited a greatly enlarged thyroid with diffuse neoplastic lesions and elevated TSH levels (Fig 1), at ages of 12 weeks. *Braf*^*CA/+*^*;TPO-Cre* mice, *Tg(LNL-Braf*^*V600E*^*)#213MM;TPO-Cre* mice, and controls exhibited serum TSH levels of 43.1 ± 56.6, 0.9 ± 0.2 and 1.0 ± 0.2 ng/ml, respectively, and thyroid weights of 122.0 ± 63.6, 8.0 ± 4.3 and 6.7 ± 1.8 mg, respectively. The lower transformation efficiency in *Tg(LNL-Braf*^*V600E*^*)****#****213MM* as compared with *Braf*^*CA*^ mice may explain our previous failure of tumor induction in *Tg(LNL-Braf*^*V600E*^*)#213MM* mice with intrathyroidal injection of Ad-TgP-Cre in our previous study [3]. Therefore, we used *Braf*^*CA*^ rather than *Tg(LNL-Braf*^*V600E*^*)****#****213MM* mice to re-evaluate the carcinogenesis of BRAF^V600E^ with our thyroid cancer model with Ad-TgP-Cre. We also examined the carcinogenesis of PTEN haploinsufficiency using *Pten*^*f/+*^ mice and *Braf*^*CA/+*^*;Pten*^*f/+*^ mice, as reduced PTEN expression alone and in combination with BRAF^V600E^ reportedly plays a significant role in the carcinogenesis of various organs [15–17].

**Fig 2:**
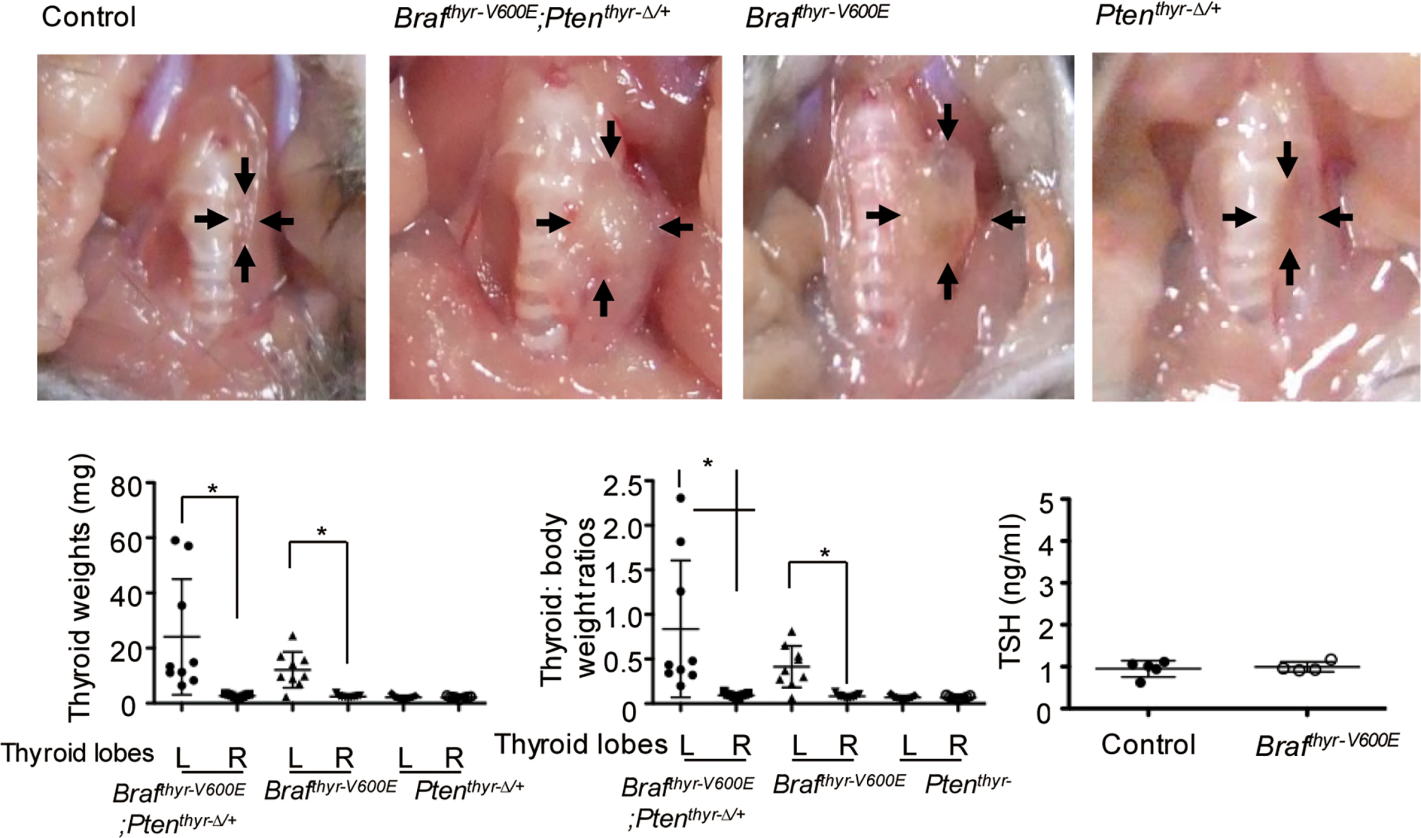
Gross appearance, thyroid weight, thyroid:body weight ratio and serum TSH concentration in the control, *Braf*^*thyr-V600E*^*;Pten*^*thyr-Δ/+*^, *Braf*^*thyr-V600E*^ and *Pten*^*thyr-Δ/+*^ mice. Adenoviral injection was performed at ~4 weeks of age. The thyroid gland and serum were collected 1 year later, and the weight and TSH concentration were determined as described in the Materials and Methods. The thyroid glands are indicated by arrows. Data are means ± S.D. (n = 5~9). *, p < 0.01.

Ad-TgP-Cre was injected into the left thyroid lobe of 4-week-old *Braf*^*CA/+*^, *Braf*^*CA/+*^*;Pten*^*f/+*^ and *Pten*^*f/+*^ mice (designated as *Braf*^*thyr-V600E*^, *Braf*^*thyr-V600E*^*;Pten*^*thyr-Δ/+*^ and *Pten*^*thyr-Δ/+*^ mice, respectively). Because it was totally unknown whether thyroid tumors developed and if so when, we decided to observe the mice either until some symptoms appeared or for 26 and 52 weeks. Because no symptom developed, the mice were sacrificed at 2 time points, as originally scheduled. The thyroid lobe was macroscopically normal in all mice at 26 weeks (data not shown), but at 52 weeks, the left lobe was enlarged in *Braf*^*thyr-V600E*^ mice (8/9) and *Braf*^*thyr-V600E*^*;Pten*^*thyr-Δ/+*^ mice (9/9), but not *Pten*^*thyr-Δ/+*^ mice (0/7) (Table 1, Fig 2). The left lobes weighed 24.0 ± 21.0 mg in *Braf*^*thyr-V600E*^*;Pten*^*thyr-Δ/+*^ mice and 12.1 ± 6.5 mg in *Braf*^*thyr-V600E*^ mice *vs*. ~ 2 mg in the right lobe of these mice (p<0.01) and also each lobe of the controls. The left lobe tended to be heavier in *Braf*^*thyr-V600E*^*;Pten*^*thyr-Δ/+*^ mice compared with *Braf*^*thyr-V600E*^ mice, but the difference was not statistically significant (Fig 2).

**Fig 3:**
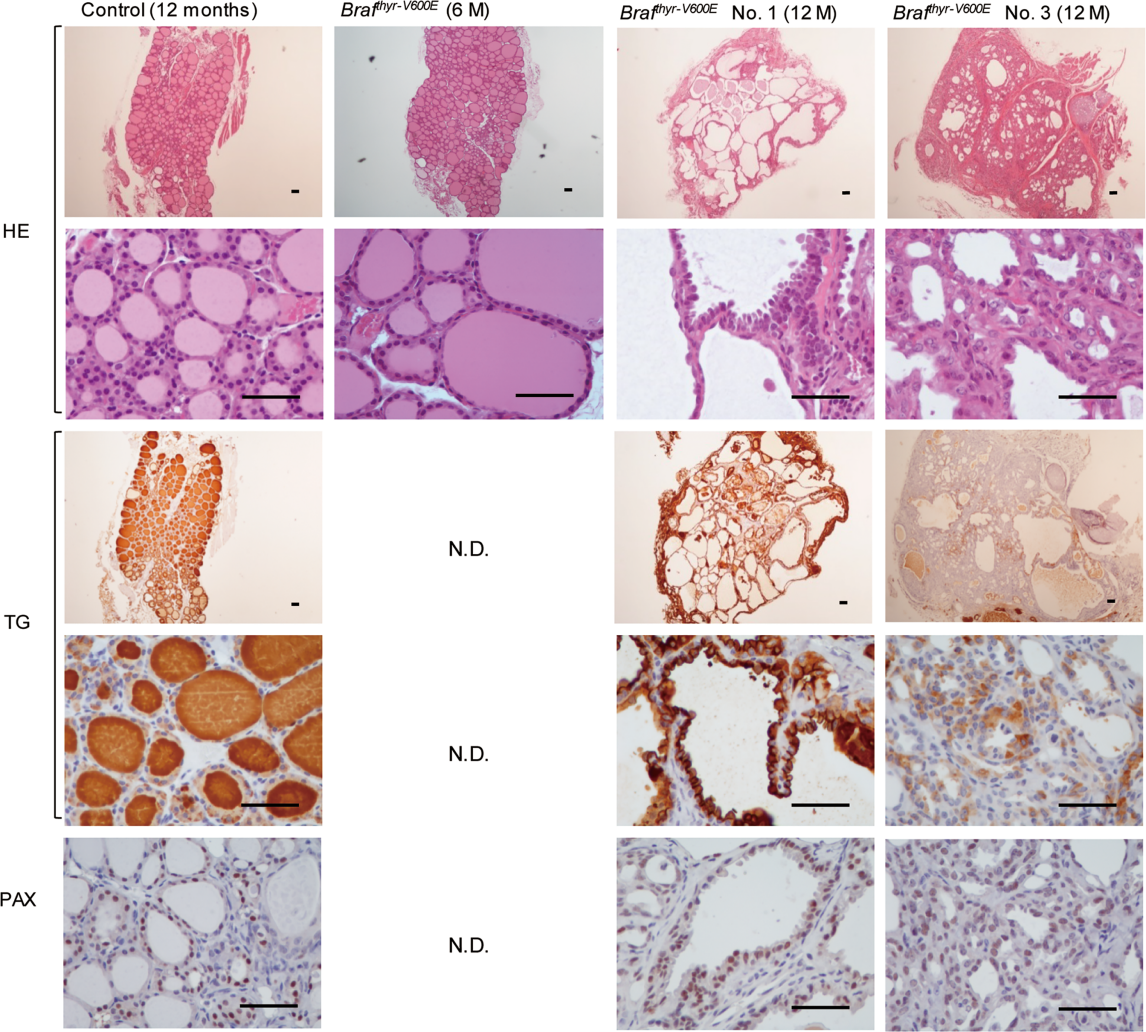
Histology of the thyroid glands from the control and *Braf*^*thyr-V600E*^ mice. The thyroid gland was removed from each mouse shown in Fig 2 and a 6 month-old *Braf*^*thyr-V600E*^ mouse, and subjected to H & E, TG and PAX staining as described in the Materials and Methods. Representative photographs of a control mouse and *Braf*^*thyr-V600E*^ mice No. 1 and No. 3 are shown. Scale bars, 50 μm.

**Fig 4:**
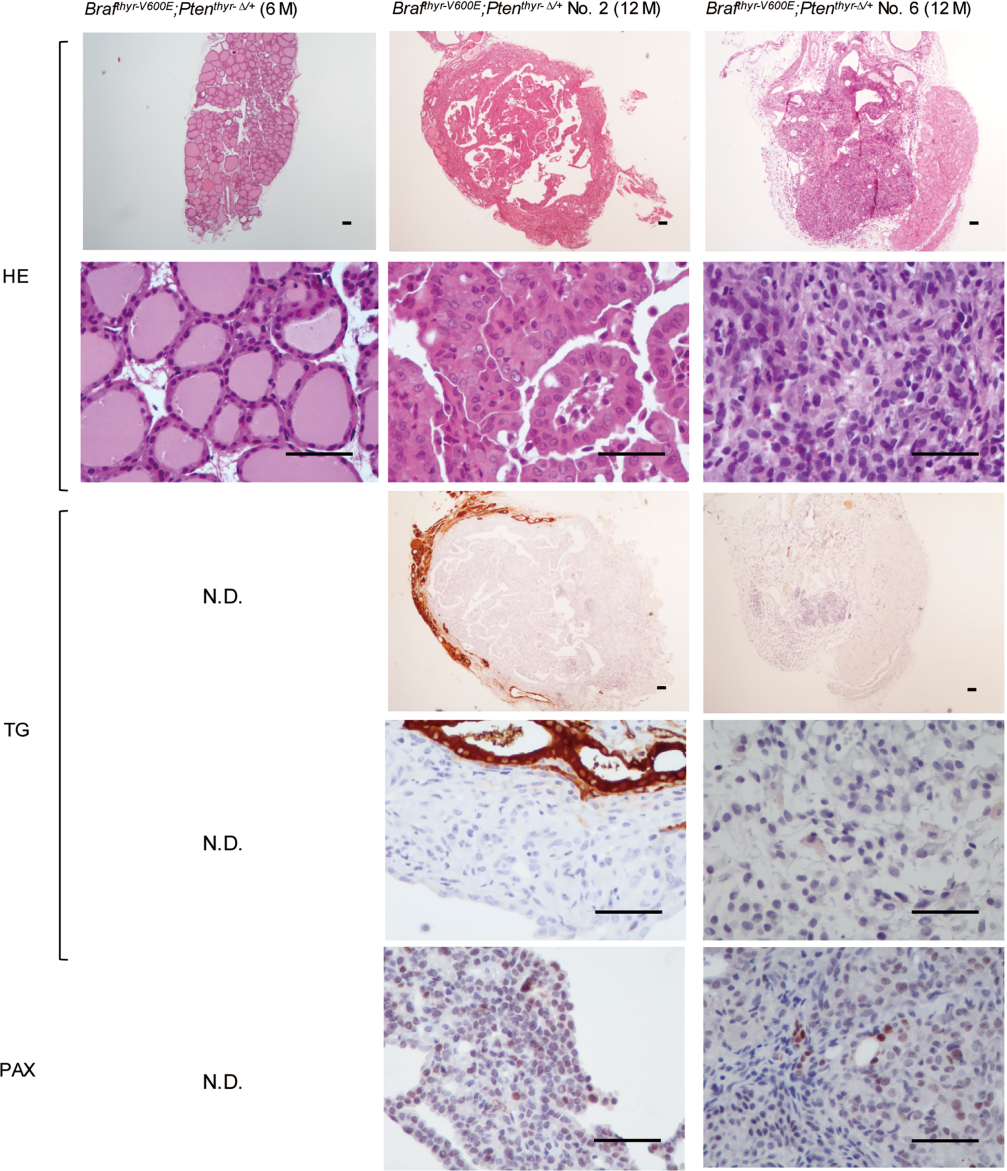
Histology of the thyroid glands from *Braf*^*thyr-V600E*^*;Pten*^*thyr-Δ/+*^ mice. The thyroid gland was removed from each mouse shown in Fig 2 and a 6-month-old *Braf*^*thyr-V600E*^*;Pten*^*thyr-Δ/+*^ mice, and subjected to H & E, TG and PAX staining as described in the Materials and Methods. Representative photographs of *Braf*^*thyr-V600E*^*;Pten*^*thyr-Δ/+*^ mice No. 2 and No. 6 are shown. Scale bars, 50 μm.

**Fig 5:**
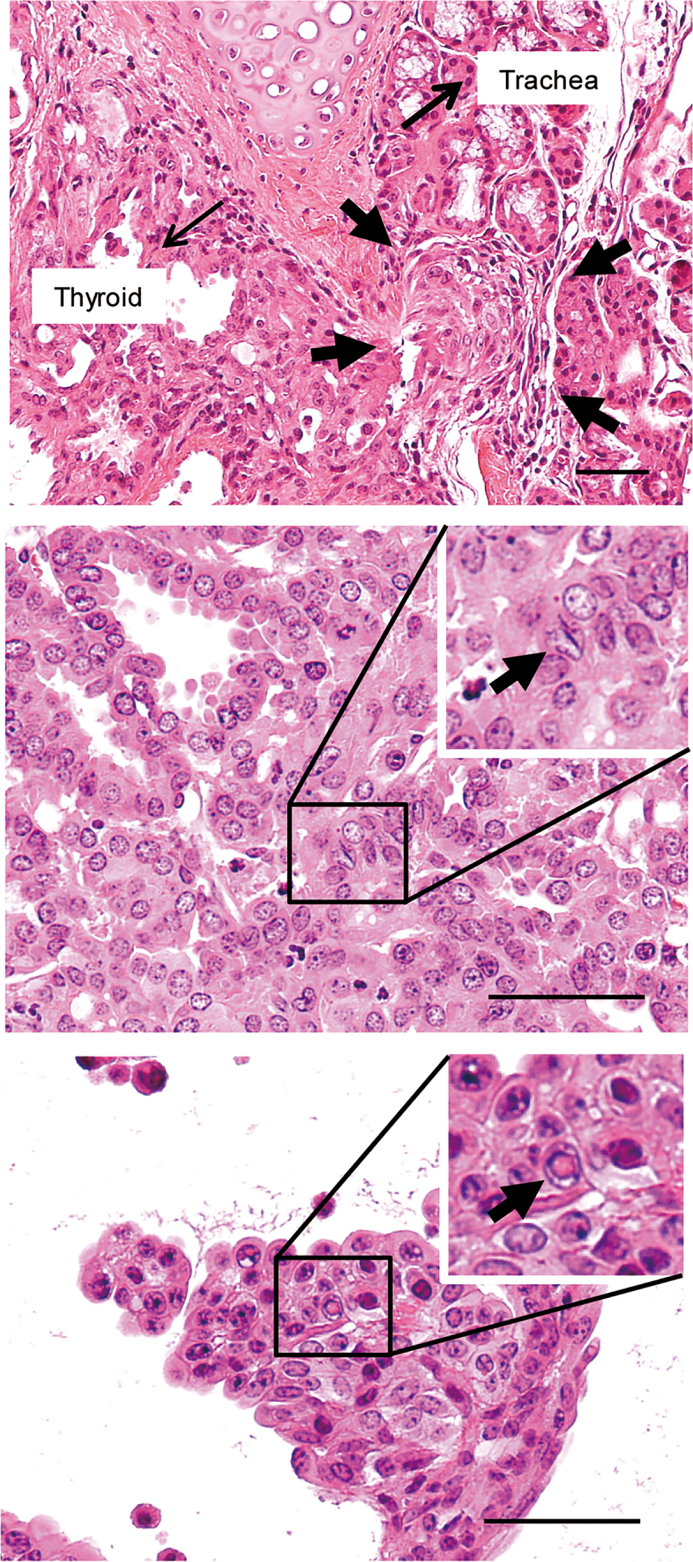
Extrathyroidal invasion and intranuclear features of thyroid cancer cells. (Upper) Invasion of the trachea (marked by the arrows). (Middle and lower) Intranuclear cytoplasmic inclusions and nuclear grooves (indicated by the arrows). Scale bars, 50 μm.

Microscopically, all of the thyroid glands obtained at 26 weeks were intact, but the tumors encompassed almost the entire thyroid gland, and almost no normal thyroid architecture was observed in the periphery of the thyroids in *Braf*^*thyr-V600E*^ and *Braf*^*thyr-V600E*^*;Pten*^*thyr-Δ/+*^ mice (Figs 3 and 4) at 52 weeks. Tumors in the majority of *Braf*^*thyr-V600E*^ mice exhibited a follicular or cribriform-like structure consisting of atypical epithelial cells with hyperchromatic swollen nuclei and no colloid formation. They also showed a hobnail pattern (represented by *Braf*^*thyr-V600E*^ mouse No. 1 in Fig 3), suggesting a loss of the tight cell to cell adhesion [18]. A hobnail pattern has not been reported in other PTC mouse models, with the exception of Rusinek and colleagues [19], who found this pattern in a small fraction of their transgenic *Tg-2HA-Braf*^*V600E*^ mice, which are similar to *Tg-Braf*^*V600E*^ mice [2]. In human PTC, this pattern of pathology is usually associated with an aggressive phenotype [20, 21]. Immunohistochemical analysis demonstrated clear TG and PAX staining of tumor cells (represented by *Braf*^*thyr-V600E*^ mouse No. 1 in Fig 3). Two tumors from *Braf*^*thyr-V600E*^ mice also contained a component of papillary structures and expressed the similar levels of PAX but decreased levels of TG (represented by *Braf*^*thyr-V600E*^ mouse No. 3 in Fig 3). In contrast, all of the tumors in *Braf*^*thyr-V600E*^*;Pten*^*thyr-Δ/+*^ mice showed predominantly papillary structures with sporadic undifferentiated areas exhibiting solid growth pattern of atypical cells with a number of mitotic figures. The nuclei were hyperchromatic, varying in size, and oval to spindle-shaped. No necrosis of single cells was observed. PAX expression was normal to low, and TG expression was low to absent (represented by No. 2 and No. 6 in Fig 4). Accompanying extrathyroidal invasion was occasionally observed (Fig 5A). Typical nuclear features of human PTC, such as intranuclear cytoplasmic inclusion and nuclear groove, were frequently observed in tumors of *Braf*^*thyr-V600E*^*;Pten*^*thyr-Δ/+*^ mice (Fig 5B and 5C).

**Fig 6:**
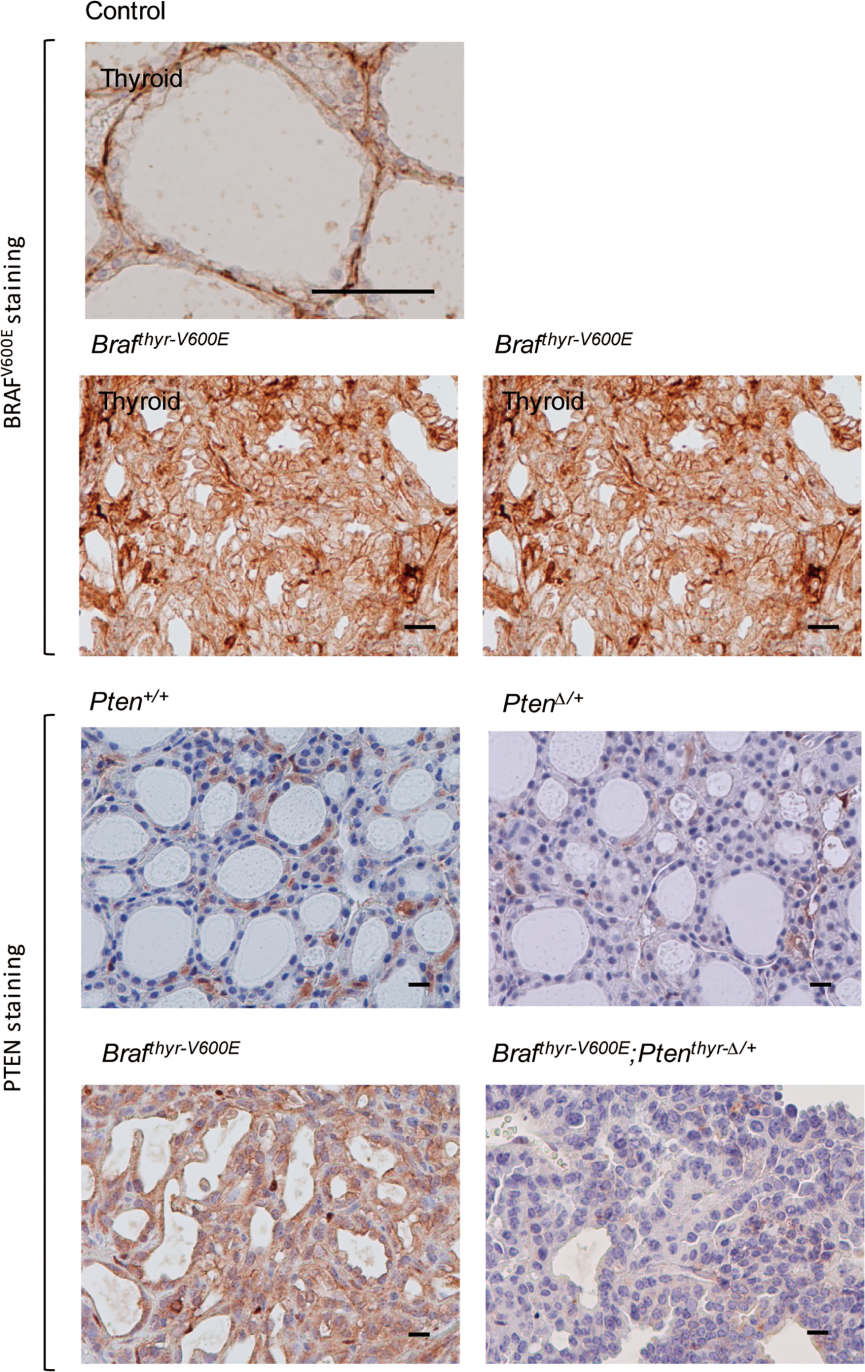
BRAF^V600E^ staining of the thyroid gland and lung tissue in control and *Braf*^*thyr-V600E*^ mice, and PTEN staining of the thyroid gland in *Pten*^*+/+*^, *Pten*^*Δ/+*^, *Braf*^*thyr-V600E*^ and *Braf*^*thyr-V600E*^*;Pten*^*thyr-Δ/+*^ mice. The thyroid gland and lung were removed in the mice from Fig 2, and subjected to BRAF^V600E^ and PTEN staining as described in the Materials and Methods. Scale bars, 50 μm.

Ad-TgP-Cre-mediated BRAF^V600E^ expression and decreased PTEN expression were confirmed by immunohistochemistry (Fig 6). Thus, BRAF^V600E^ was expressed in thyroid cancer but not in the normal thyroid, although the basement membrane-like region stained non-specifically stained in the normal thyroid glands. Expression of PTEN was clearly observed in the thyroids of *Pten*^*+/+*^ and *Braf*^*thyr-V600E*^ mice, but barely detectable in *Pten*^*Δ/+*^ and *Braf*^*thyr-V600E*^*;Pten*^*thyr-Δ/+*^ mice.

Thyroid tumors exhibiting (i) typical nuclear features of human PTC such as intranuclear cytoplasmic inclusions and nuclear grooves and/or (ii) invasion of the extrathyroidal tissues surrounding the thyroid glands were readily diagnosed as cancers. Some tumors in *Braf*^*thyr-V600E*^ mice not exhibiting these features were also judged as cancers, because they had malignant characteristics such as structural atypia, including cribriform-like, papillary, and solid growth of atypical follicular cells with hyperchromatic swollen nuclei, which occasionally showed a hobnail pattern.

**Fig 7:**
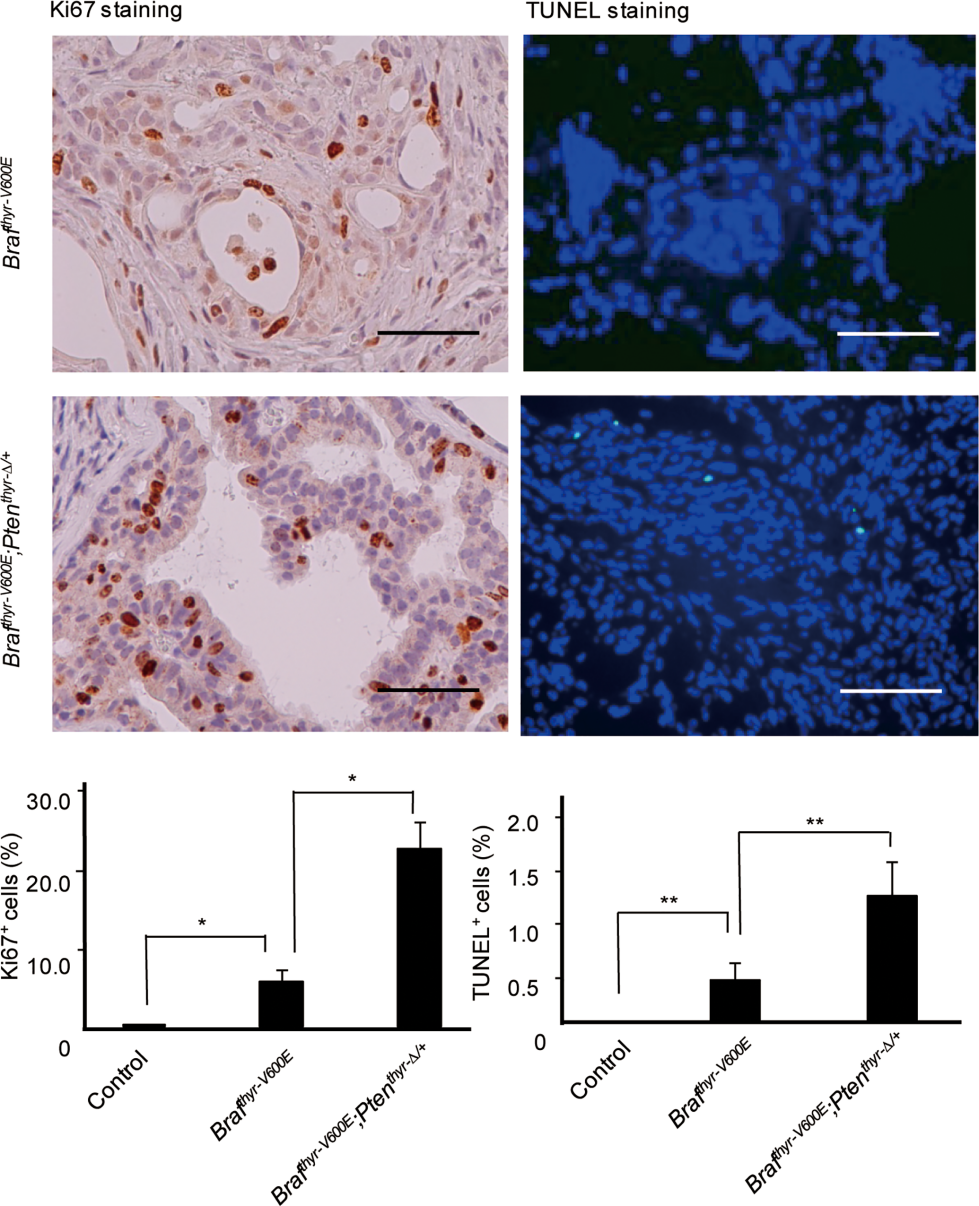
Ki67 and TUNEL staining of the thyroid gland from the control, *Braf*^*thyr-V600E*^ and *Braf*^*thyr-V600E*^*;Pten*^*thyr-Δ/+*^ mice. The thyroid gland from each mouse in Fig 2 was subjected to Ki67 and TUNEL staining as described in the Materials and Methods. Data are means ± S.D. (n = 5~9). *, p < 0.01; **, p < 0.05. Scale bars, 50 μm.

**Fig 8:**
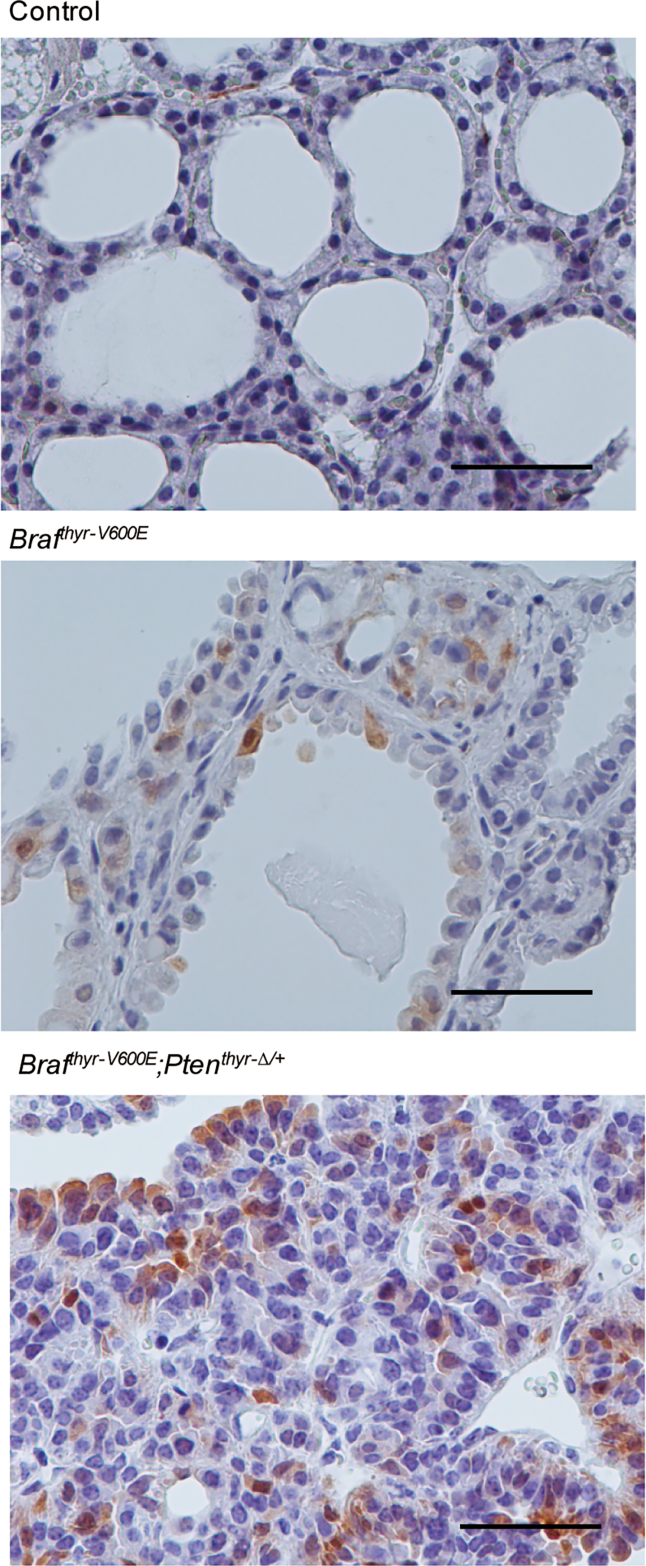
Phosphorylated ERK staining of the thyroid gland from control, and *Braf*^*CA/w*^ and *Braf*^*thyr-V600E*^*;Pten*^*thyr-Δ/+*^ mice. The thyroid gland from each mouse in Fig 2 was subjected to pERK staining as described in the Materials and Methods. Scale bars, 50 μm.

Higher cell proliferation indices determined by Ki67 staining (22.5 ± 10.2 *vs*. 5.6 ± 4.6) were compensated by higher cell death rates as determined by TUNEL staining (1.1 ± 0.9 *vs*. 0.4 ± 0.4) in *Braf*^*thyr-V600E*^*;Pten*^*thyr-Δ/+*^ mice as compared with *Braf*^*thyr-V600E*^ mice (Fig 7), which likely explains the non-significant difference in tumor sizes between the 2 mouse groups (Fig 2). Although the staining intensity seemed stronger in *Braf*^*thyr-V600E*^*;Pten*^*thyr-Δ/+*^ than *Braf*^*thyr-V600E*^ mice in immunohistochemical analysis of phosphorylated ERK, intra- and inter-tumoral heterogenous staining made quantitative comparison of expression in both groups difficult. Representative photographs are shown in Fig 8.

**Fig 9:**
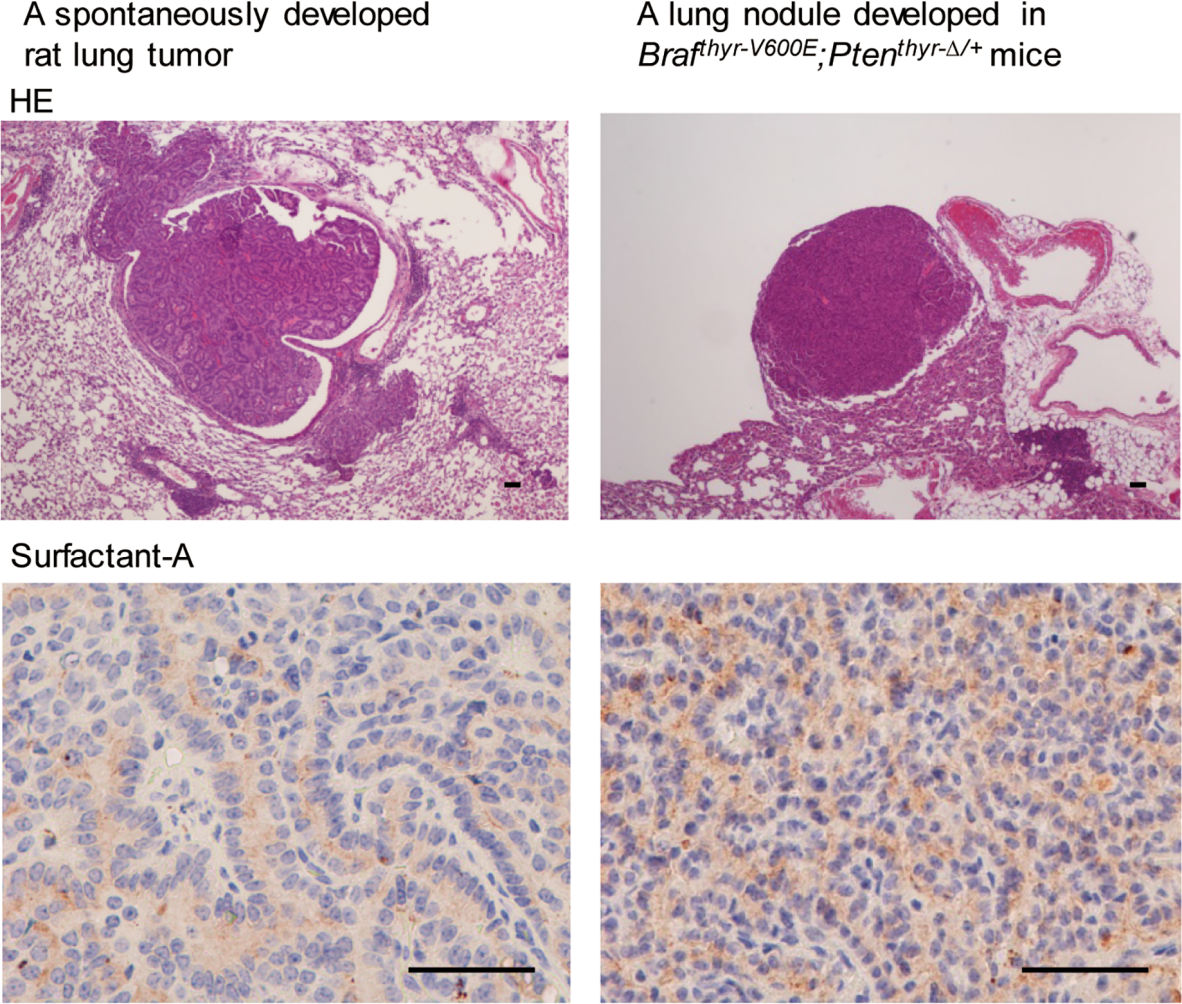
H & E and surfactant protein-A staining in a spontaneously developed rat lung tumor [22] (as a positive control) and in a lung nodule developed in *Braf*^*thyr-V600E*^*;Pten*^*thyr-Δ/+*^ mouse. Scale bars, 50 μm.

Macroscopic lung nodules were observed in 2 of 9 *Braf*^*thyr-V600E*^ and 6 of 9 *Braf*^*thyr-V600E*^*;Pten*^*thyr-Δ/+*^ mice. BRAF^V600E^ expression in these nodules (Fig 6) excluded the possibility of the spontaneously arisen primary lung tumors, but negative staining for TG and PAX (data not shown) did not provide convincing evidence that these nodules were metastases. Although Ad-TgP-Cre-mediated BRAF^V600E^ expression was very unlikely even if adenovirus had disseminated systemically, because the *Tg* promoter we used in this study is exclusively thyroid-specific and has been widely and successfully used for many genetically engineered mice (e.g., *Tg-Braf*^*V600E*^) [2], we found that these nodules were positive for surfactant protein-A (Fig 9), which is reportedly expressed in BRAF^V600E^-induced lung adenomas [6, 16]. A spontaneously developed rat lung tumor [22] also stained positive.

**Fig 10:**
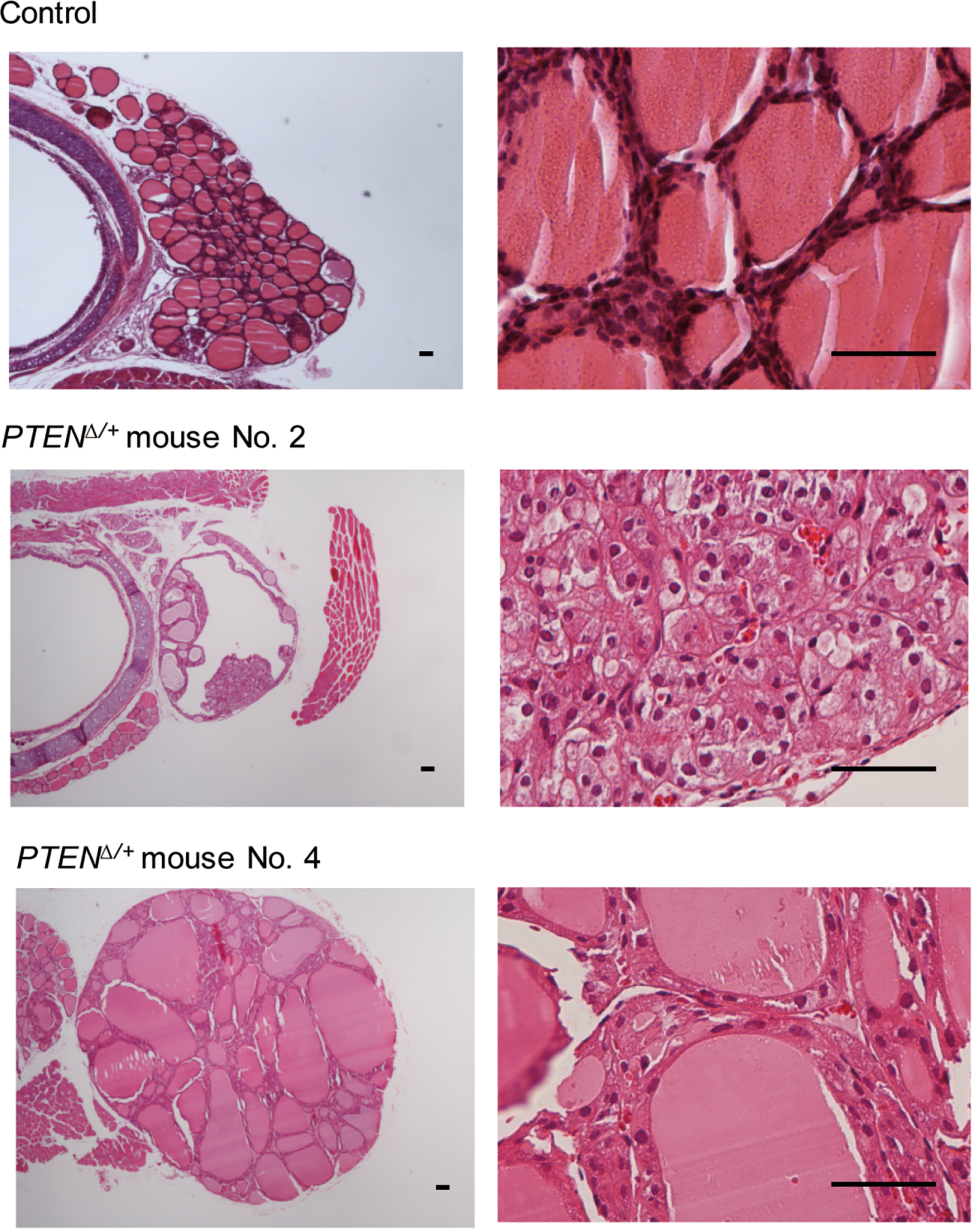
Thyroid histology of *Pten*^*Δ/+*^ mice. Mice were sacrificed at ~6~8 months of age due to development of tumors in other organs. Representative photographs of *Pten*^*Δ/+*^ mice No. 2 and No. 4 are shown. Scale bars, 50 μm.

Finally, despite the absence of tumor development in *Pten*^*thyr-Δ/+*^ mice, most *Pten*^*Δ/+*^ mice developed thyroid hyperplasia/adenoma by the age of 6 to 8 months (Table 1, Fig 10). These mice were sacrificed during this time period because tumor had developed in other organs.

## Discussion

Although we previously reported the insufficiency of postnatal expression of BRAF^V600E^ for thyroid cancer development in mice [3], in the present study, we re-evaluated this issue using a different genetically engineered mouse model (*i*.*e*., *Braf*^*CA*^). As BRAF^V600E^ is frequently found in sporadic thyroid cancers in euthyroid subjects, BRAF^V600E^ should be expressed in a small fraction of thyroid cells (ideally in a single cell, but it is currently not possible experimentally) after birth under physiologic TSH levels. In this regard, our experimental design—that is, intrathyroidal injection of Ad-TgP-Cre into one side of the thyroid lobes of genetically engineered mice harboring the *lox*P sequences—is likely ideal. The feasibility of adenovirus-mediated Cre gene transfer to temporally and spatially control Cre expression has been well demonstrated [23, 24]. In the present study, we clearly showed that thyroid cancers *did* develop in Ad-TgP-Cre-injected *Braf*^*CA*^ mice, indicating that postnatal expression of BRAF^V600E^ alone under physiologic TSH levels is sufficient for thyroid cancer development. Similar preliminary results were reported by McFadden *et al* (see Fig. S1H in ref. [10]).

Our previous failure with *Tg(LNL-Braf*^*V600E*^*)* mice [3] appeared to be attributable to a lower efficiency of Cre-mediated DNA recombination, although we cannot exclude the other possibilities that the different genetic backgrounds (B6C3 in *Tg(LNL-Braf*^*V600E*^*) vs*. B6 in *Braf*^*CA*^) and/or different promoters (CAG promoter *vs*. the endogenous *Braf* promoter) could have affected our previous results. Different recombination frequencies of distinct alleles have been reported [25]. Presumably, the frequency of transformation of BRAF^V600E^-expressing normal, differentiated (*i*.*e*., TG-expressing) thyroid cells into malignant cells is extremely low.

The *Braf*^*CA*^*;TPOCreER* mouse model with tamoxifen reported by McFadden *et al*. may also be ideal, although the TSH levels increased slightly (<10 fold) [10]. However, thyroid cancers developed several weeks after administration of tamoxifen in their model, in a sharp contrast to the present study, in which thyroid cancers were only detectable 1 year (not 6 months) after adenovirus injection. It is unclear whether the slight increase in TSH promoted tumorigenesis in their model. In this regard, fine dose-response experiments may be necessary to find the appropriate concentration of tamoxifen to induce thyroid cancer on one hand while maintaining physiologic TSH levels on the other.

Significant increases in TSH levels (up to 500 fold) have been noted in other models [2, 4, 9, 10]. As elevated TSH is known to induce thyroid enlargement and sometimes promote tumorigenesis by itself [26], there is no doubt that elevated TSH has substantially affected the results obtained with the above-mentioned mouse models of thyroid cancer with marked TSH elevation. However, the significance of low TSH levels for thyroid tumorigenesis is controversial. On one hand, *Tg-Braf*^*V600E*^*;Tshr*^*-/-*^ *mice* [27] and *LSL-Braf*^*V600E*^*;TPO-Cre;Tshr*^*-/-*^ mice [4], both of which are unresponsive to TSH stimulation due to a lack of TSH receptor expression, can develop thyroid cancers, albeit less aggressive, but, on the other hand, transplantation of thyroid cancers developed in *LSL-Braf*^*V600E*^*;TPO-Cre* mice (with high TSH levels) into nude or syngeneic immuno-competent mice (with normal TSH levels) leads to regression and senescence [28].

Regarding the question as to how many mutations are required for full development of differentiated thyroid cancer, recent studies using human samples show that number of non-synonymous mutations in exomes is ~0.4/Mb [29–31], and the number of mutations among 341 cancer-related genes in PTC is reportedly 1 ± 1 (median ± interquartile range) [30, 32]. Thus, similar to pediatric cancer and leukemia, thyroid cancer is associated with a very low number of mutations, suggesting that a single or perhaps only a few mutations are sufficient for thyroid cancer to develop. In our model, however, the possibility cannot be excluded that other mutations occurred during the 1-year observation period.

BRAF^V600E^ was first discovered in malignant melanoma, but later also found to be present in benign nevi, which seldom progress to melanoma unless additional mutations occur [33]. In accordance with this observation, in mouse experiments, BRAF^V600E^ alone cannot induce melanoma, but it can in combination with PTEN loss or activating PI3KCA mutations [16, 34]. Concurrent mutations in BRAF and diminished PTEN expression are common in human melanomas [34]. Similar data were also reported in lung adenocarcinoma and prostate cancer in genetically engineered mice [17, 35]. Of interest, in contrast to thyroid cancer, melanoma and lung cancer are among cancers with a high number of mutations [29, 31].

The combination of BRAF^V600E^ and reduced PTEN expression tended to induce larger and more undifferentiated thyroid cancers in our study, and these data were similar to those in *LSL-Braf*^*V600E*^*;Pten*^*f/f*^*;TPO-Cre* mice in which PTC rapidly progressed to poorly differentiated thyroid cancers as compared with *LSL-Braf*^*V600E*^*;TPO-Cre* mice [36] and also to those in *Thyro*::*CreER;Braf*^*CA/+*^*;Pik3ca*^*lat-1047R/+*^ mice, which developed anaplastic cancers as compared with *Thyro*::*CreER;Braf*^*CA/+*^ mice [37]. Although the mutations in *Pten* gene are not common [38], reduced expression of PTEN due to hypermethylation is frequently detected even in differentiated thyroid cancers [39].

Tumorigenesis associated with PTEN loss by itself is well known in human Cowden syndrome, in which a germline loss-of-function mutation in the *PTEN* gene induces thyroid multinodular goiter and adenoma [40]. Experimentally, the tumorigenesis of prenatal PTEN loss in the mouse thyroid gland was clearly shown by Yeager *et al*. using *Pten*^*L/L*^*;TPO-Cre* mice [15]. Thus, similar to the *Pten*^*Δ/+*^ mice used in our study, the majority of mice in the 129Sv genetic background developed well-circumscribed follicular adenomas and nodular hyperplasia, often characterized by increased cellularity and mitotic figures at 8 to 10 months of age. However, no thyroid tumors were observed in *Pten*^*f/+*^ mice injected with Ad-TgP-Cre in our study. These data clearly indicate that the tumorigenic potential of reduced PTEN expression differs between the prenatal and postnatal periods.

We interpret our data on lung tumors as showing that adenovirus injected into the thyroid lobes leaked, disseminated systemically, and reached the lung, where BRAF^V600E^ was expressed aberrantly from the *Tg* promoter, even when the volume of adenovirus injected was low (1 μl) and highly thyroid specific *Tg* promoter was used. Thus, one of the limitations of our study is the leakiness of locally injected adenovirus as well as leakiness of the *Tg* promoter. Our model is therefore not suitable for study of metastasis. Only 2 reports of lung metastasis have been reported, one by Rusinek *et al*. using transgenic *Tg-2HA-Braf*^*V600E*^ mice [19] and the other by McFadden using *TPOCreER;Braf*^*CA/+*^*;p53*^*LSL-R270H/+*^ mice [10]. Another limitation is that we cannot completely exclude the possible effect of adenovirus-induced inflammation and/or disruption of local tissue architecture on cancer development in our experimental setting.

In conclusion, using our mouse model with Ad-TgP-Cre, we show that postnatal expression of BRAF^V600E^ alone under physiologic TSH levels is sufficient for development of thyroid cancer and that simultaneous reduced expression of PTEN tends to promote tumor growth and de-differentiation. It will be of interest in the future to compare the differences/similarities of thyroid cancers associated with postnatal *vs*. prenatal expression of BRAF^V600E^. Our data also indicate that the effects of BRAF^V600E^ expression and reduced PTEN expression differ between the prenatal *vs*. postnatal periods. Thus, unlike BRAF^V600E^, the tumorigenic potential of PTEN depends on a prenatal reduction in expression.
